# Precipitation and Species Composition Mediate Litter Mixing Decomposition Effects in Arid Desert Regions

**DOI:** 10.3390/plants15111759

**Published:** 2026-06-05

**Authors:** Tingting Xie, Lishan Shan, Haixia Wang, Zhuopeng Fan, Hongyong Wang

**Affiliations:** College of Forestry, Gansu Agricultural University, Lanzhou 730070, China; xieting1026@126.com (T.X.);

**Keywords:** precipitation change, litter decomposition, non-additive effects, desert plants

## Abstract

Mixed-species litters exhibit decomposition characteristics distinct from those of monospecific litters and are highly sensitive to precipitation changes in arid desert regions. However, the effects of altered precipitation on mixed-litter decomposition in these ecosystems remain poorly understood. Fresh leaf litter of *Reaumuria soongarica*, *Salsola passerina* and *Nitraria sphaerocarpa* was collected from arid desert habitats. We investigated decomposition differences among three monospecific litters and four mixed-species litters under different precipitation treatments. For both monospecific and mixed litters, increased precipitation significantly accelerated mass loss and nitrogen release through the entire experimental period. Compared with the natural precipitation treatment (CK), litter mass loss increased by 10.97–18.07%, while nitrogen (N) release increased by 4.64–7.96% after 12 months of decomposition. By contrast, decreased precipitation only significantly reduced carbon release. Increased precipitation did not shift the non-additive effects on mass loss from antagonistic to synergistic; it only weakened the antagonistic effects. Mixing effects on nutrient release varied among litter mixtures. Two-species mixtures exhibited stronger antagonistic effects than three-species mixtures, and increased precipitation moderately enhanced the synergistic effects in two-species mixtures. In conclusion, precipitation change altered litter decomposition in desert plants but had limited effects on litter-mixture interactions.

## 1. Introduction

Litter is a crucial component of terrestrial ecosystems and serves as an important link between soil and vegetation. Its decomposition is a key process governing carbon and nutrient cycling among plants, soil, and the atmosphere [1,2]. In natural terrestrial ecosystems, different plant species produce litter with distinct physical and chemical characteristics [3], resulting in significant litter-mixing effects. These effects may synergistically promote or antagonistically inhibit litter decomposition and nutrient release, thereby regulating ecosystem material cycling [4]. Precipitation is one of the primary environmental factors influencing litter decomposition, and changes in precipitation inevitably alter the decomposition dynamics of mixed-species litter [5]. This phenomenon is particularly important in arid regions, where ecosystem processes are highly sensitive to precipitation fluctuations. Therefore, understanding how mixed-litter decomposition responds to precipitation change is essential for predicting the effects of climate change on nutrient cycling under shifts in vegetation composition in terrestrial ecosystems.

Climate change has altered global precipitation patterns [6], thereby exerting profound effects on litter decomposition processes [7]. Previous studies have reported inconsistent responses of litter decomposition to precipitation change. Increased precipitation generally accelerates litter decomposition and nutrient release, whereas reduced precipitation often suppresses decomposition rates [8]. However, some studies have reported no significant effects of precipitation change on decomposition processes [9]. Similarly, the responses of non-additive effects in mixed-litter decomposition to precipitation change vary among vegetation types and ecosystems. For instance, Duan et al. [10] reported that non-additive effects were not significantly influenced by climatic or environmental variation. In contrast, studies conducted in alpine grasslands of northern Tibet showed that increased precipitation enhanced synergistic effects [11], whereas drought weakened such effects [12]. Other studies have reported contrasting patterns, with drought either reducing [13] or enhancing [14] non-additive effects. These inconsistencies highlight the need for region-specific studies on mixed-litter decomposition under altered precipitation regimes to improve predictions of ecosystem nutrient cycling under climate change.

In arid desert regions, *R. soongarica*, *S. passerina*, and *N. sphaerocarpa* commonly coexist as dominant shrub species and form mixed plant communities [15]. The decomposition of their litter plays an important role in maintaining soil fertility in these water-limited ecosystems. Previous studies have shown that mixed-species litter derived from these species decomposes more slowly than monospecific litter and exhibits pronounced non-additive effects [16]. However, whether precipitation change can alter the decomposition dynamics and litter-mixing effects of these litter mixtures remains unclear. To address this knowledge gap, we aimed to evaluate how precipitation change regulates litter decomposition and litter-mixing effects in arid desert ecosystems. We hypothesized that increased precipitation would enhance litter mass loss and nutrient release by alleviating moisture limitation during decomposition, whereas non-additive litter-mixing effects would remain primarily constrained by litter chemical traits. Therefore, increased precipitation was expected to weaken antagonistic effects without necessarily shifting them toward synergistic effects. We further hypothesized that three-species mixtures would exhibit weaker antagonistic effects than two-species mixtures due to greater chemical complementarity among litter types.

## 2. Results

### 2.1. Litter Mass Loss and Nutrient Release

**Figure 1:**
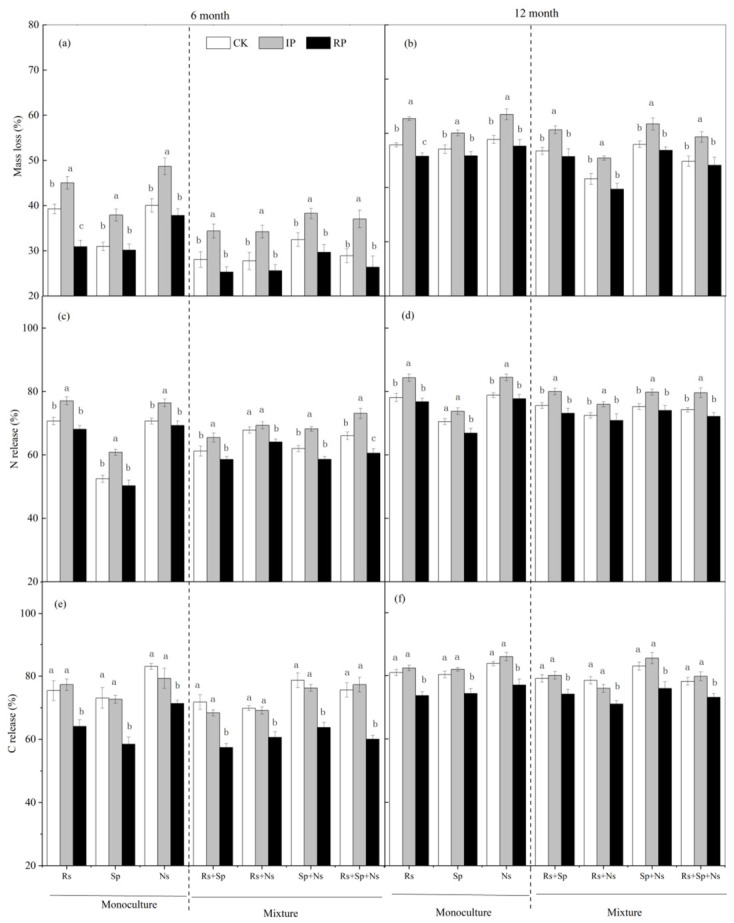
Mass loss (**a**,**b**), N release (**c**,**d**) and C release (**e**,**f**) of monoculture and mixed litters over one-year decomposition period. Data are mean values (*n* = 6) and error bars represent the standard error. Different lowercase letters indicate significant differences among different precipitation treatments (*p* < 0.05). Rs, *Reaumuria soongarica*, Sp, *Salsola passerina* and Ns, *Nitraria sphaerocarpa*

Precipitation treatment, specific composition, and sampling date all significantly affected litter mass loss, N release, and C release (Table 1). The interaction between species composition and sampling date was also significant for all three variables. Across the 12-month decomposition period, the 30% increased precipitation treatment (IP) significantly promoted mass loss compared with the other two treatments, whereas the 30% reduced precipitation treatment (RP) showed no significant inhibitory effect regardless of species composition (Figure 1a,b). Relative to the natural precipitation treatment (CK), litter mass loss under the IP treatment increased by 14.65–28.14% and 10.97–18.07% after 6 and 12 months of decomposition, respectively.

Nitrogen release was significantly enhanced under the IP treatment compared with CK, whereas the RP treatment did not significantly reduce N release. In contrast, increased precipitation did not significantly affect C release, while reduced precipitation significantly decreased C release across all litter types. Under the IP treatment, N release increased by 2.11–15.93% and 4.64–7.96% after 6 and 12 months of decomposition, respectively (Figure 1c,d). By comparison, C release under the RP treatment decreased by 13.18–20.60% and 6.23–9.52% after 6 and 12 months of decomposition, respectively (Figure 1e,f).

### 2.2. Mixing Effects on Litter Mass Loss and Nutrient Release

**Figure 2:**
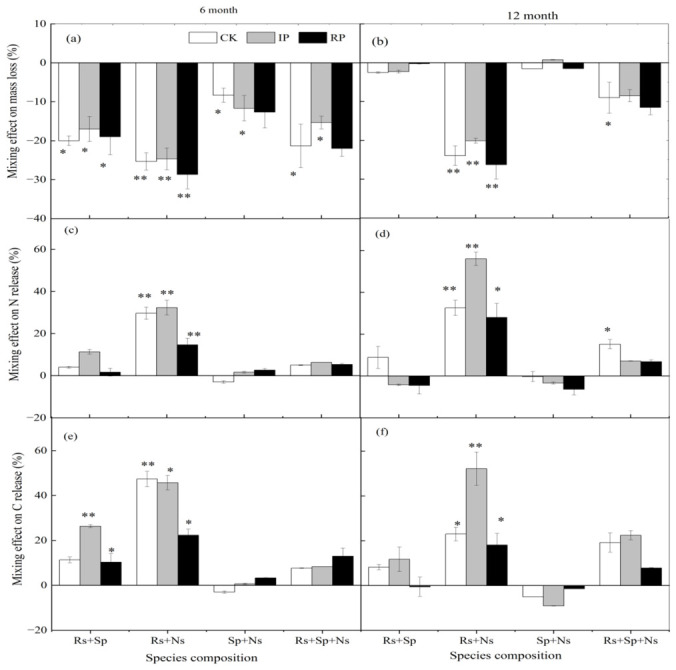
Mixing effects of mass loss (**a**,**b**) and net nutrient release (**c**–**f**) for litter mixtures over one-year decomposition period. Data are mean values (*n* = 6) and error bars represent the standard error. The significant differences between the litter mixing effects and zero are indicated by * (*p* < 0.05) and ** (*p* < 0.01). Rs, *Reaumuria soongarica*, Sp, *Salsola passerina* and Ns, *Nitraria sphaerocarpa.*

Precipitation treatment and sampling date significantly affected litter-mixing effects on mass loss, N release and C release, while species composition significantly affected litter-mixing effects only on mass loss and C release (Table 1). The interaction between species composition and sampling date was also significant. Across all precipitation treatments, litter mixtures exhibited antagonistic effects on mass loss after 6 months of decomposition. Increased precipitation weakened these antagonistic effects, whereas reduced precipitation partially alleviated them. At the 12 months of decomposition, only the mixture of *R. soongorica* + *N. sphaerocarpa* (Rs + Ns) exhibited antagonistic effects on mass loss under all three precipitation treatments, although increased precipitation also weakened the magnitude of these antagonistic effects. In contrast, additive effects on mass loss were observed in the other three mixtures, except for the *R. soongorica* + *S. passerine* + *N. sphaerocarpa* (Rs + Sp + Ns) under the natural precipitation treatment (CK) (Figure 2a,b).

For the Rs + Ns mixture, synergistic effects on N and C release were detected under all three precipitation treatments after both 6 and 12 months of decomposition, and these synergistic effects were significantly enhanced under 30% increased precipitation treatment (IP). In the *R. soongorica* + *S. passerine* (Rs + Sp) mixture, synergistic effects on C release were observed under both the 30% increased precipitation (IP) and 30% reduced precipitation (RP) treatments after 6 months of decomposition (Figure 2c–f).

### 2.3. Individual Species Response of Mass Loss to Mixture

**Figure 3:**
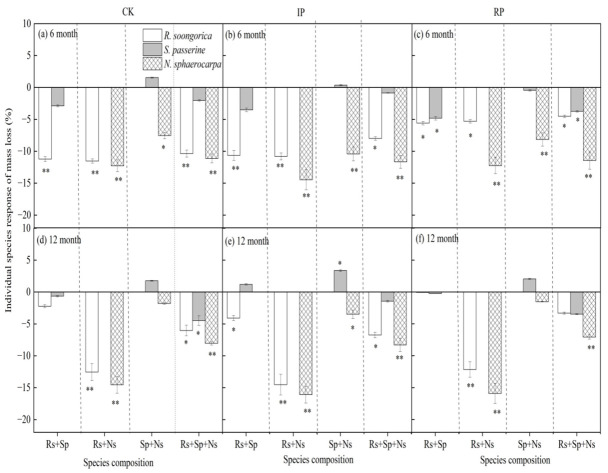
Individual species responses of mass loss to mixture. (**a**–**c**) show the individual species response of mass loss under CK, IP and RP treatment after 6 months of decomposition, whereas (**d**–**f**) represent the individual species response of mass loss under CK, IP and RP treatment for 12 months of decomposition. Data are mean values (*n* = 6) and error bars represent the standard error. The significant differences between individual species responses and zero are indicated by * (*p* < 0.05) and ** (*p* < 0.01). Rs, *Reaumuria soongarica*, Sp, *Salsola passerina* and Ns, *Nitraria sphaerocarpa.*

After 6 months of decomposition under the natural precipitation treatment (CK), *R. soongorica* exhibited negative responses to litter mixing in all three mixed-species litter treatments (Rs + Sp, Rs + Ns and Rs + Sp + Ns), with reductions ranging from −10.38% to −11.54%. These negative responses were weakened under both the 30% increased precipitation treatment (IP; −8.00% to −10.81%) and the 30% reduced precipitation treatment (RP; −4.52% to −5.59%) (Figure 3a–c). Similarly, *N. sphaerocarpa* exhibited negative responses in the Rs + Ns, Sp + Ns and Rs + Sp + Ns mixtures, with reductions ranging from −7.55% to −12.29% under CK. These negative responses were further strengthened under both the IP treatment (−10.42% to −14.47%) and RP treatment (−8.16% to −12.25%) (Figure 3a–c). After 12 months of decomposition, both *R. soongorica* and *N. sphaerocarpa* continued to exhibit negative responses in the Rs + Ns and Rs + Sp + Ns mixtures under CK. These negative responses were further enhanced under IP treatment. In contrast, the negative response of *R. soongorica* disappeared under the RP treatment in the Rs + Sp + Ns mixture (Figure 3d–f). By comparison, *S. passerina* exhibited a positive response in the Sp + Ns mixture (Figure 3e).

### 2.4. Litter-Mixing Effects on Decomposition

**Figure 4:**
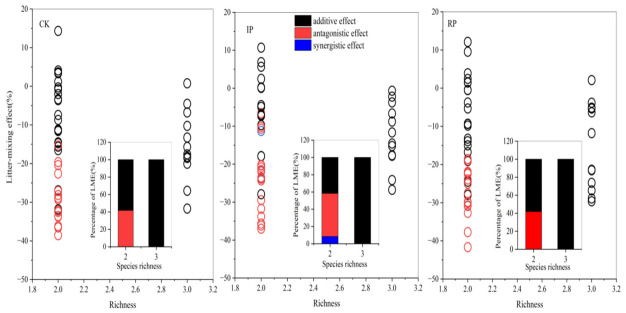
Litter-mixing effects of litter mass loss at different species-richness levels throughout the decomposition period under the control (CK), 30% increased precipitation (IP), and 30% reduced in precipitation (RP) treatments. Blue, red and black symbols represent synergistic, antagonistic and additive effects, respectively. The inset panels show the proportions of different litter-mixing effects under the two species-richness levels.

Throughout the one year of decomposition period, among the two-species mixtures, 41.67% exhibited antagonistic effects and 58.33% exhibited additive effects under the natural precipitation treatment (CK) and the 30% reduced precipitation treatment (RP). Under the 30% increased precipitation treatment (IP), 8.34% of the mixtures exhibited synergistic effects, 50% exhibited antagonistic effects, and 41.66% presented additive effects (Figure 4). For the three-species mixtures, higher species richness promoted stronger additive effects on decomposition under all three precipitation treatments. The mean litter-mixing effects of both two-species and three-species mixtures under the three precipitation treatments were significantly greater than zero (*p* < 0.05).

## 3. Discussion

### 3.1. Effect of Mixed Litter on Mass Loss and Nutrient Release Under Different Precipitation

Previous studies have reported inconsistent effects of precipitation on litter decomposition, which may be attributed to differences in litter types, microbial activity, and environmental conditions [12,17,18,19,20]. In arid regions, precipitation-induced increases in soil moisture generally stimulate the abundance and activity of soil biota, thereby promoting litter decomposition [21]. Consistent with our first hypothesis, increased precipitation significantly enhanced the mass loss of both monospecific and mixed-species litter throughout the experimental period. Such positive decomposition responses are plausibly linked to improved soil moisture conditions (Figure S1) which is widely recognized to facilitate soil enzyme activity. Notably, soil enzyme activity was not directly measured in the present study. As such, the potential linkage between moisture variation and enzyme-mediated decomposition in this study is a plausible interpretation. This result is supported by our supplementary soil moisture data and well-documented moisture–enzyme relationships from previous studies [21].

Precipitation regimes can alter nutrient release by regulating soil microbial activity [22]. However, our results revealed contrasting responses of C and N release to precipitation change, partially contradicting our first hypothesis. Nitrogen release responded significantly to increased precipitation, whereas C release decreased only under reduced precipitation. These findings suggest N release is more sensitive to increased precipitation, consistent with the observed patterns of litter mass loss. By contrast, reduced precipitation likely decreased soil moisture (Figure S1) and potentially suppressed the activity of enzymes associated with litter carbon decomposition (Figure S2).

### 3.2. Effect of Different Precipitation on Litter Mixture Decomposition

Non-additive litter-mixing effects on decomposition are widely observed in natural ecosystems [23]. In the present study, antagonistic effects on mass loss were detected in all litter mixtures after 6 months of decomposition under natural precipitation conditions, whereas after 12 months, antagonistic effects persisted only in the Rs + Ns mixture. This pattern is consistent with the findings of Zhang et al. [24], who reported that antagonistic effects may persist during long-term decomposition. Such persistent antagonistic interactions were likely associated with relatively similar leaf nitrogen contents but substantial differences in lignin concentrations among the three species. The transfer and accumulation of recalcitrant compounds, particularly lignin, within litter mixtures may prolong inhibitory interactions during decomposition [25], This mechanism may explain why antagonistic effects remained evident in the Rs + Ns mixture after 12 months, as this mixture likely retained relatively high lignin content [26].

Partially inconsistent with our first hypothesis, increased precipitation weakened antagonistic effects but did not shift them toward synergistic effects. One possible explanation is that the duration of the precipitation treatment was insufficient to substantially increase soil microbial abundance or enzyme activity during the early decomposition stage [27]. This interpretation is consistent with previous studies showing that decomposition responses to precipitation change are strongly time-dependent in semi-arid shrubland ecosystems [28]. In addition, persistent antagonistic interactions may have been regulated primarily by litter chemical traits [25,26]. Changes in precipitation may alter litter physicochemical characteristics and generate legacy effects that maintain relatively high concentrations of recalcitrant compounds, thereby limiting the transition from antagonistic to synergistic interactions. Previous studies have shown that non-additive litter-mixing effects are jointly regulated by litter traits, climatic conditions, and decomposition stage [29]. Synergistic interactions are generally more likely to occur in litter mixtures with low litter quality or under strong soil-fauna activity, whereas antagonistic interactions tend to persist when litter contains abundant inhibitory secondary metabolites, such as phenolic compounds [30]. These findings suggest that litter chemical traits may serve as a key regulatory factor modulating decomposition dynamics in the present study, even though increased precipitation partially weakened antagonistic interactions.

Under natural precipitation conditions, synergistic effects on nutrient release were detected only in the Rs + Ns mixture after both 6 and 12 months of decomposition, whereas additive effects predominated in the remaining mixtures. This result suggests that litter-mixing effects on nutrient release differed from those on litter mass loss [31]. Increased precipitation enhanced synergistic nutrient release in Rs + Ns mixture and shifted additive effects on C release toward synergistic interactions in the Rs + Sp, partially contradicting our first hypothesis. Although precipitation can modify the magnitude of non-additive effects on nutrient release, it does not override the fundamental regulatory role of litter chemical traits. Species-specific differences in water-retention capacity may have contributed to the observed synergistic nutrient-release patterns [14]. However, these hydrological characteristics are themselves closely associated with litter physical and chemical traits, including litter structure and wax content. Taken together, our findings support the reasonable interpretation that litter traits, rather than precipitation alone, are the primary driver shaping decomposition dynamics in these arid desert ecosystems. This interpretation is also consistent with our key finding that increased precipitation weakened, but did not eliminate antagonistic litter-mixing effects, further implying the prominent regulatory role of litter chemical traits in regulating decomposition processes [14].

### 3.3. Effect of Species Richness on Litter Mixture Decomposition

Numerous studies have demonstrated that litter decomposition is regulated by both litter species identity and species diversity [4,32]. Some studies have suggested that species composition, rather than species richness, primarily determines the non-additive effects of litter mixtures [33,34], whereas others have reported that higher species richness may increase the likelihood of synergistic interactions during decomposition [35]. Consistent with our second hypothesis, antagonistic effects were predominantly observed in the two-species mixtures, whereas additive effects were more common in the three-species mixtures across all precipitation treatments. One possible explanation for this pattern is the difference in chemical complementarity between litter mixtures with different species-richness levels. In the two-species mixtures, the relatively similar initial nutrient contents of the component litter types may have resulted in overlapping substrate utilization and enzyme requirements during decomposition. Such overlap could intensify competitive interactions among decomposers communities, thereby promoting antagonistic interactions during litter decomposition. By contrast, the three-species mixtures likely exhibited greater chemical heterogeneity and resource complementarity, which may have alleviated competition among decomposers. As chemical diversity increased, these inhibitory interactions were progressively weakened, ultimately resulting in additive decomposition patterns [24].

## 4. Materials and Methods

### 4.1. Study Area

This study was conducted in the desert ecosystem observation site in the Linze Inland River basin, located in the central Hexi Corridor of Northwest China (39°41′ N, 100°12′ E). The mean annual average precipitation and mean annual temperature in the study area are 117 mm and 7.6 °C, respectively, whereas the mean annual evaporation reaches 2390 mm. The dominant shrub species include *R. soongarica*, *S. passerina*, and *N. sphaerocarpa*, while the herbaceous species mainly include *Suaeda glauca*, *Halogeton arachnoideus*, and *Eragrostis minor* Host. Additional information regarding this desert ecosystem is provided in the study by Xie et al. [8].

### 4.2. Precipitation Design

Based on meteorological data from the past 50 years in the study area, precipitation fluctuations generally varied within ±30% [36]. Therefore, three precipitation treatments were designed: natural precipitation (CK), 30% increased precipitation (IP), and 30% reduced precipitation (RP). A random block design was adopted, consisting of six blocks, each containing the three precipitation treatments (3 × 6 = 18 plots).

To achieve the precipitation-manipulation treatments, V-shaped rainout shelters were used in the precipitation-reduction plots. The shelters were constructed from transparent acrylic plates with low yellowing indices and high ultraviolet radiation transmittance. The ratio of the blocked area covered by the V-shaped grooves to the total plot area represented the precipitation-reduction ratio (30%). To maintain air circulation and minimize microclimatic disturbances within the plots, each rainout shelter consisted of a white steel frame supporting the V-shaped acrylic grooves. Each rainout shelter covered a 2 × 2 m plot and was installed 1 m above the soil surface. The shelters were positioned at a 10° southward slope to facilitate the drainage of intercepted precipitation into collection sinks. Following each precipitation event, the collected water from each RP plot was evenly added within 8 h to the corresponding IP plot. To maintain light transmittance, dust accumulated on the acrylic grooves was removed every 5–7 days. During the entire experimental period, a total of 15 precipitation events occurred, with cumulative precipitation reaching 79.2 mm.

### 4.3. Decomposition Design and Sampling

Fresh leaf litter of *R. soongorica* (Rs), *S. passerina* (Sp) and *N. sphaerocarpa* (Ns) was collected from the desert ecosystem observation site in October 2022. The collected litter was first air-dried for one week and then six subsamples (10 g each) of air-dried samples were oven-dried at 65 °C to constant mass to determine dry mass, water content, and the initial concentrations of C, N, P, and lignin and cellulose (Table 2).

Three monospecific litter treatments (Rs, Sp and Ns) and four mixed-species litter treatments (Rs + Sp, Rs + Ns, Sp + Ns, and Rs + Sp + Ns) with equal mass proportions were established in this experiment. For each treatment, 15 g of litter was placed into 10 × 10 cm litterbags with a mesh size of 0.5 mm. In total, 252 litterbags were deployed across 18 experimental plots, corresponding to three precipitation treatments × seven litter compositions × six replicates × two sampling dates. The litterbags were fixed to the soil surface using iron wire to prevent displacement by wind.

After 6 and 12 months of decomposition, the litterbags for each litter treatment in each plot were collected. Large arthropods and attached debris were carefully removed using a brush. The litter samples were then oven-dried at 80 °C to a constant mass and weighed to determine litter mass loss. Subsequently, the dried samples were ground to determine element concentrations. Total organic C concentrations were determined using the K_2_Cr_2_O_7_ oxidation method, whereas total N concentrations were measured using the Kjeldahl method [37].

### 4.4. Statistical Analysis

Litter mass loss and nutrient release were calculated as follows:

$$\mathrm{Mass}\;\mathrm{loss}(\%)=\frac{M_{0}-M_{t}}{M_{0}}\times 100$$

$$\mathrm{Nutrient}\;\mathrm{release}(\%)=\frac{M_{0}\times C_{0}-M_{t}\times C_{t}}{M_{0}\times C_{0}}\times 100$$

where *M_t_* is the remaining oven-dry mass (g) at sampling time, *M*_0_ is the initial oven-dry mass (g), *C*_0_ is the initial element concentration (mg g^−1^), and *C_t_* is the element concentration (mg g^−1^) at the sampling time.

Relative mixing effects (RME) were calculated as follows:

$$RME(\%)=\frac{\left(O-E\right)}{E}\times 100$$

where *O* is the observed mass loss or nutrient release of the litter mixtures, and *E* is expected litter mass loss or nutrient release, calculated as the weighted mean value of the corresponding monocultures based on their proportions with each mixture [11]. Paired *t*-tests were used to determine whether RMEs differed significantly from zero under the three precipitation treatments. Litter-mixture interactions were classified as additive (no significant difference between observed and expected values), synergistic (observed values significantly higher than expected values), or antagonistic (observed values significantly lower than expected values) [38].

The Individual species response to litter mixing was calculated as: Individual species response (%) = Mixture − Alone, where Mixture and Alone represent the mass loss (%) of component species decomposing in litter mixture and monocultures, respectively. One-sample *t*-tests were used to determine whether individual species responses differed significantly from zero and negative when values were lower than zero. The effects of precipitation treatment, species composition, sampling date and their interactions on litter mass loss and nutrient release were evaluated using three-way ANOVA. One-way ANOVA was used to test differences in litter mass loss and nutrient release among precipitation treatments.

## 5. Conclusions

This study investigated the effects of precipitation change and species composition on litter decomposition over a 12-month decomposition period. Increased precipitation significantly promoted litter mass loss and N release, whereas reduced precipitation decreased C release, but did not significantly suppress litter mass loss or N release. A key finding of this study is that litter mixtures generally exhibited antagonistic interactions with respect to litter mass loss. Notably, increased precipitation only weakened these antagonistic interactions but did not fully shift them toward synergistic interactions. Regarding nutrient release, the Rs + Ns mixture was the only litter combination that exhibited synergistic effects on both N and C release, and these synergistic interactions were further enhanced under increased precipitation. By contrast, the remaining litter mixtures mainly exhibited additive interactions that were not significantly influenced by precipitation treatment. With respect to species composition, the two-species mixtures exhibited stronger antagonistic interactions than the three-species mixtures. Importantly, although precipitation modification substantially altered the magnitude of litter-mixing interactions, it failed to overturn the prevailing antagonistic interaction structure of litter mass loss observed across most mixture combinations. Overall, precipitation change and species composition jointly regulated litter decomposition, nutrient release, and litter-mixing interactions in this desert ecosystem. These findings advance our understanding of how altered precipitation regimes modulate litter decomposition and non-additive mixing effects in arid desert environments.

## Figures and Tables

**Table 1:** Results (F-values) of three-way ANOVA indicating effects of precipitation treatment (P), species composition (C), and sampling date (D) on decomposition dynamics and mixing effects. Asterisks indicate significant differences (* *p* < 0.05 and ** *p* < 0.01) and ns is no significant differences.

			Mass Loss		N Release		C Release	
		df	F	*p*-Value	F	*p*-Value	F	*p*-Value
Decomposition dynamics	P	2	146.01 **	<0.0001	161.91 **	<0.0001	159.59 **	<0.0001
	C	6	46.85 **	<0.0001	89.58 **	<0.0001	22.05 **	<0.0001
	D	1	1669.02 **	<0.0001	818.79 **	<0.0001	250.10 **	<0.0001
	P * C	12	0.84 ns	0.609	1.1 ns	0.371	0.74 ns	0.711
	C * D	6	7.75 **	<0.0001	10.32 **	<0.0001	3.81 **	0.002
	P * D	2	0.51 ns	0.602	2.67 ns	0.075	14.93 **	<0.0001
	P * C * D	12	0.22 ns	0.997	0.98 ns	0.471	0.96 ns	0.494
Mixing effects	P	2	64.20 **	<0.0001	86.42 **	<0.0001	155.14 **	<0.0001
	C	3	24.17 **	<0.0001	2.3 ns	0.09	24.58 **	<0.0001
	D	1	876.05 **	<0.0001	483.49 **	<0.0001	151.73 **	<0.0001
	P * C	6	0.13 ns	0.992	1.64 ns	0.157	1.47 ns	0.153
	C * D	3	10.39 **	<0.0001	8.49 **	<0.0001	30.59 **	<0.0001
	P * D	2	0.31 ns	0.732	1.99 ns	0.148	8.33 **	<0.001
	P * C * D	6	0.09 ns	0.997	1.45 ns	0.218	2.31 *	0.013

**Table 2:** Initial chemical composition of litters (mean ± SE). Different lowercase letters within the same column indicate significant differences among litter types (*p* < 0.05)

Species	C (mg g^−1^)	N (mg g^−1^)	P (mg g^−1^)	C/N	C/P	Cellulose (mg g^−1^)	Lignin (mg g^−1^)
*R. soongarica*	498.4 ± 27.2 ^a^	5.03 ± 0.8 ^a^	3.54 ± 0.2 ^a^	99.68 ± 23.8 ^a^	142.40 ± 6.6 ^a^	178.93 ± 14.6 ^a^	208.23 ± 13.0 ^a^
*S. passerina*	451.5 ± 30.1 ^a^	3.34 ± 0.6 ^b^	2.23 ± 0.1 ^b^	136.81 ± 10.9 ^b^	205.22 ± 11.4 ^b^	173.70 ± 13.5 ^a^	175.53 ± 12.7 ^b^
*N. sphaerocarpa*	472.9 ± 51.2 ^a^	4.81 ± 0.3 ^b^	2.92 ± 0.1 ^b^	98.32 ± 8.7 ^b^	161.95 ± 10.2 ^b^	195.35 ± 16.2 ^a^	164.28 ± 11.4 ^b^

## Data Availability

The original contributions presented in this study are included in the article/Supplementary Material. Further inquiries can be directed to the corresponding author.

## Supplementary Materials

The following supporting information can be downloaded at: https://www.mdpi.com/article/10.3390/plants15111759/s1. Figure S1: soil moisture of monocultures and mixtures litter over one-year decomposition period. Data are mean values (*n* = 6) and error bars represent the standard deviation. Figure S2: Cellulase activity of monocultures and mixtures litter over one-year decomposition period. Data are mean values (*n* = 6) and error bars represent the standard deviation.
