# Supplementary material for: Precipitation and Species Composition Mediate Litter Mixing Decomposition Effects in Arid Desert Regions
Source: Plants (Basel). 2026 Jun 5;15(11):1759. doi: 10.3390/plants15111759 (PMC13258916; doi:10.3390/plants15111759)
Supplement: Supplementary file 1 [file plants-15-01759-s001.zip › plants-4235636-supplementary.pdf]

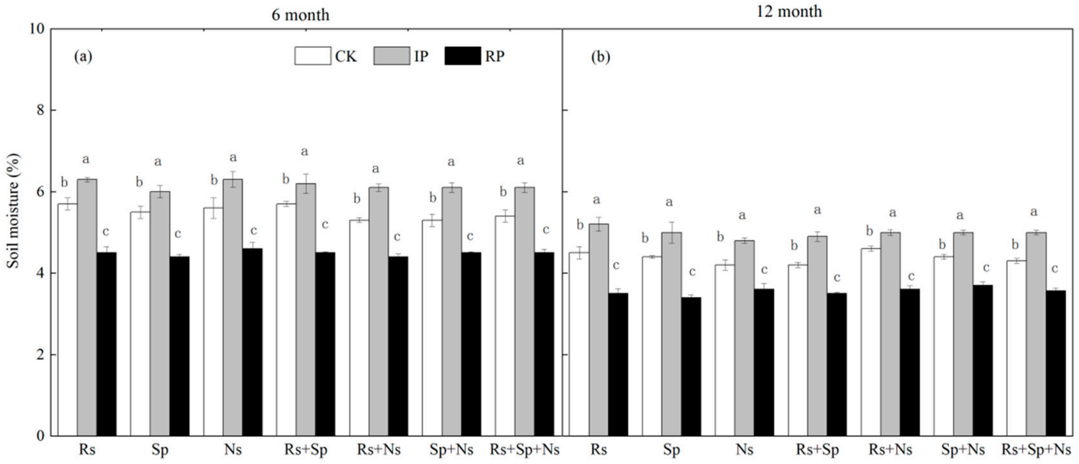

Figure S1 soil moisture of monocultures and mixtures litter over one-year decomposition period. Data are mean values (n = 6) and error bars represent the standard deviation.

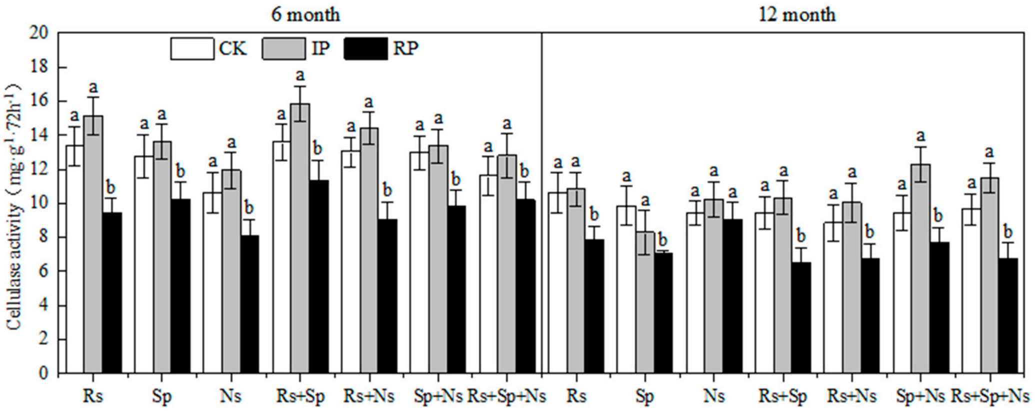

Figure S2 Cellulase activity of monocultures and mixtures litter over one-year decomposition period. Data are mean values (n = 6) and error bars represent the standard deviation.
